# Supplementary material for: Fragment ion intensity prediction improves the identification rate of non-tryptic peptides in timsTOF
Source: Nat Commun. 2024 May 10;15:3956. doi: 10.1038/s41467-024-48322-0 (PMC11087512; doi:10.1038/s41467-024-48322-0)
Supplement: Supplementary file 1 — Supplementary Information [file 41467_2024_48322_MOESM1_ESM.pdf]

Supplementary Figures

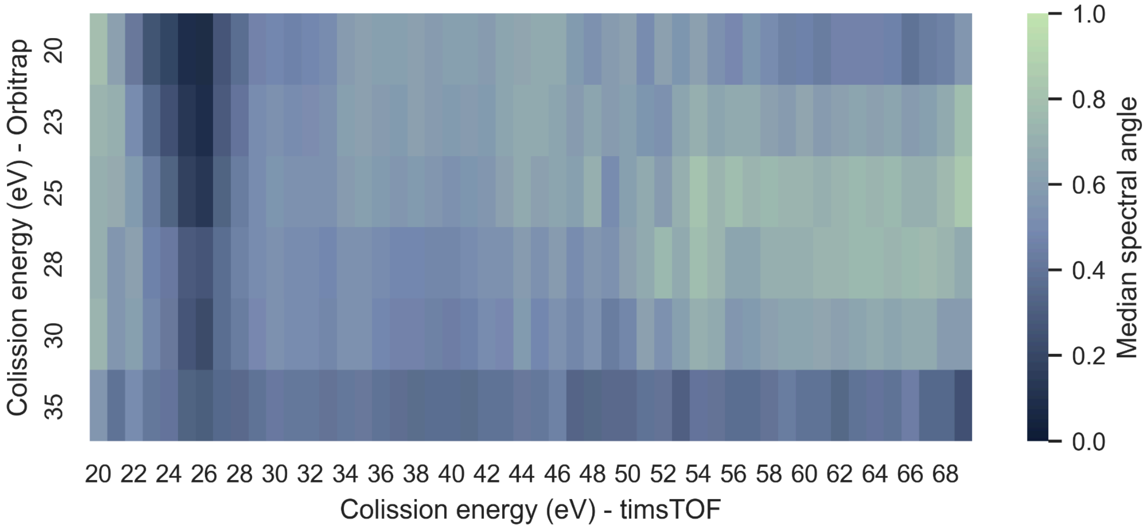

Supplementary Figure 1. The dissimilarity between MS/MS spectra generated by timsTOF and Orbitrap instruments for the same peptides was assessed by calculating the normalized spectral contrast angle between all MS/MS spectra with the same peptide sequence and charge state. The Orbitrap spectra originated from the hold-out set of Wilhelm et al. [1]. The collision energies do not seem to translate between the two machines. Source data are provided as a source data file.

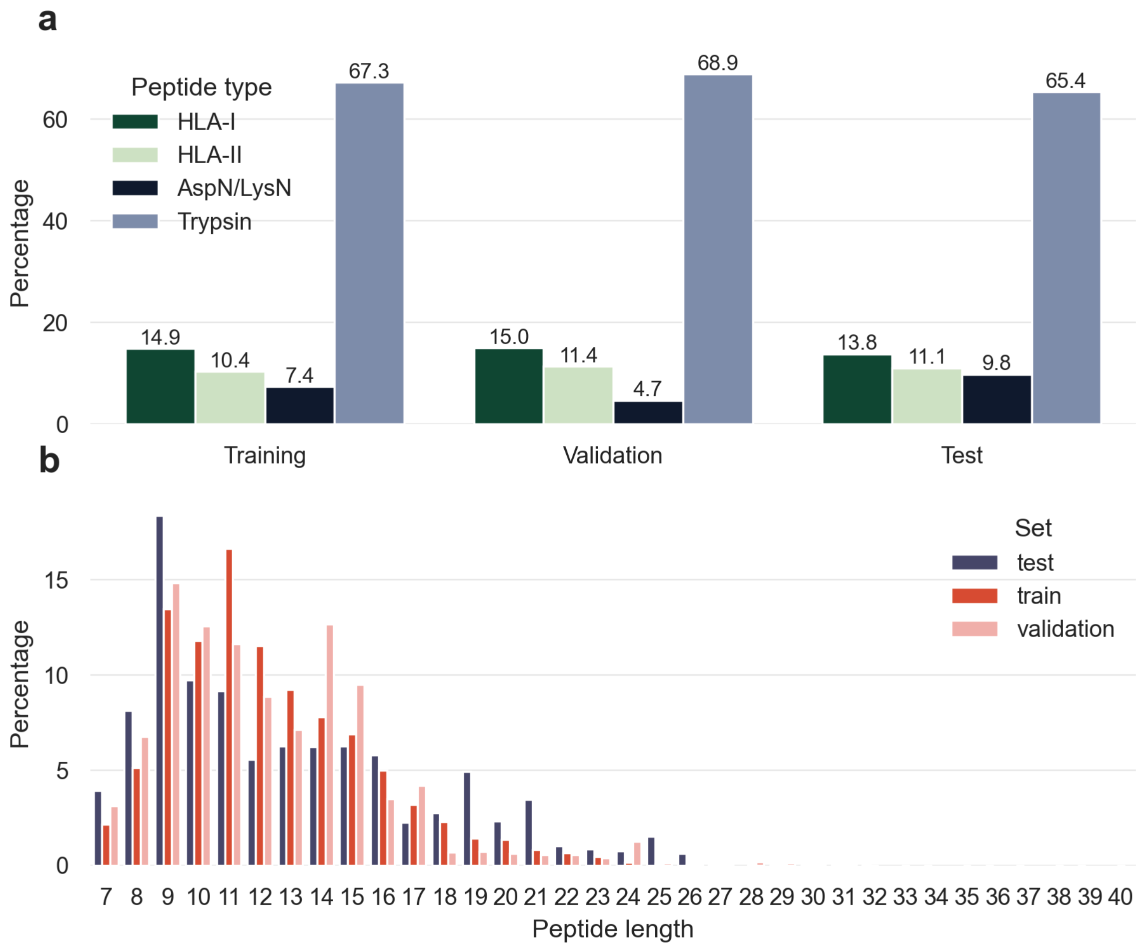

Supplementary Figure 2. Descriptive analysis of the compiled data. a Distribution of the different peptide types across the training, validation, and test sets. **b** Distribution of the peptide lengths across the training, validation, and test sets.

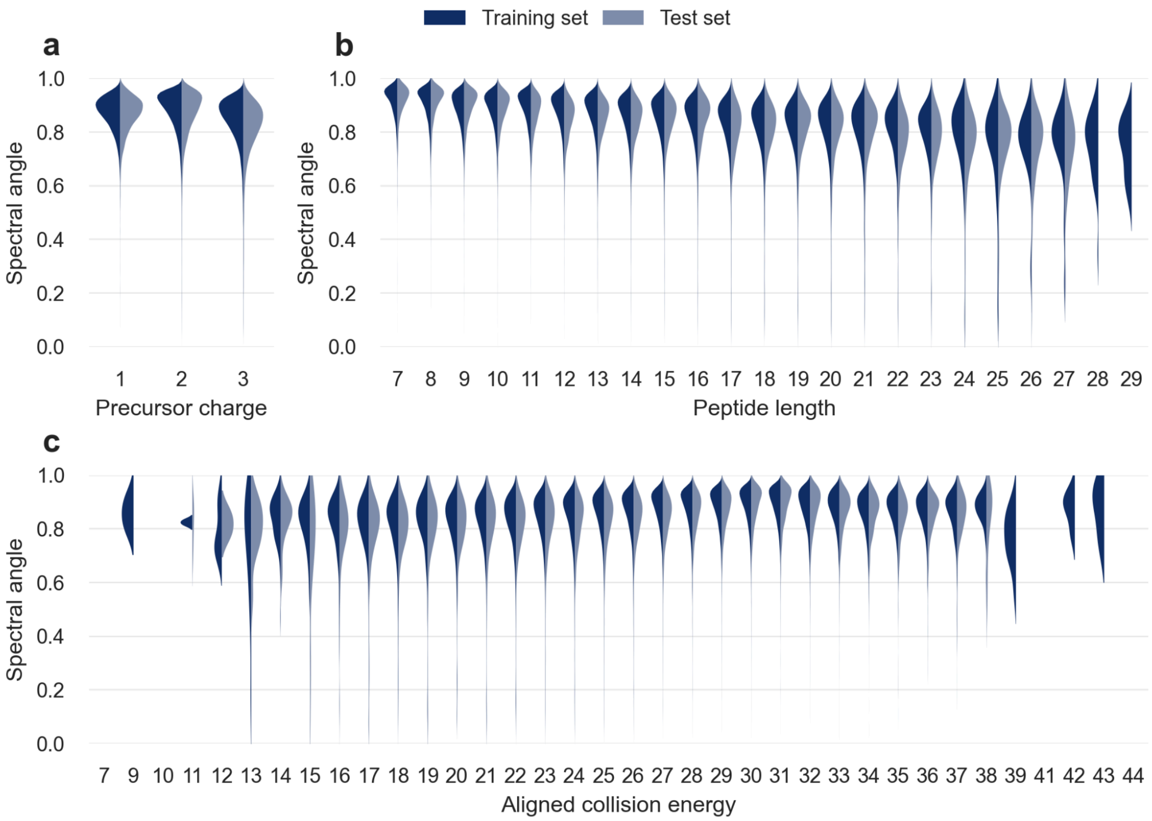

Supplementary Figure 3. The performance of the timsTOF Prosit 2023 model was assessed using asymmetric violin plots depicting the normalized spectral contrast angle between predicted and observed spectra in both the training and test sets. The model performed well across different precursor charges (**a**) and a wide range of collision energies (**c**) The model did show a moderate influence of the peptide length (**b**) on the predicted fragment ion intensities. Source data are provided as a source data file.

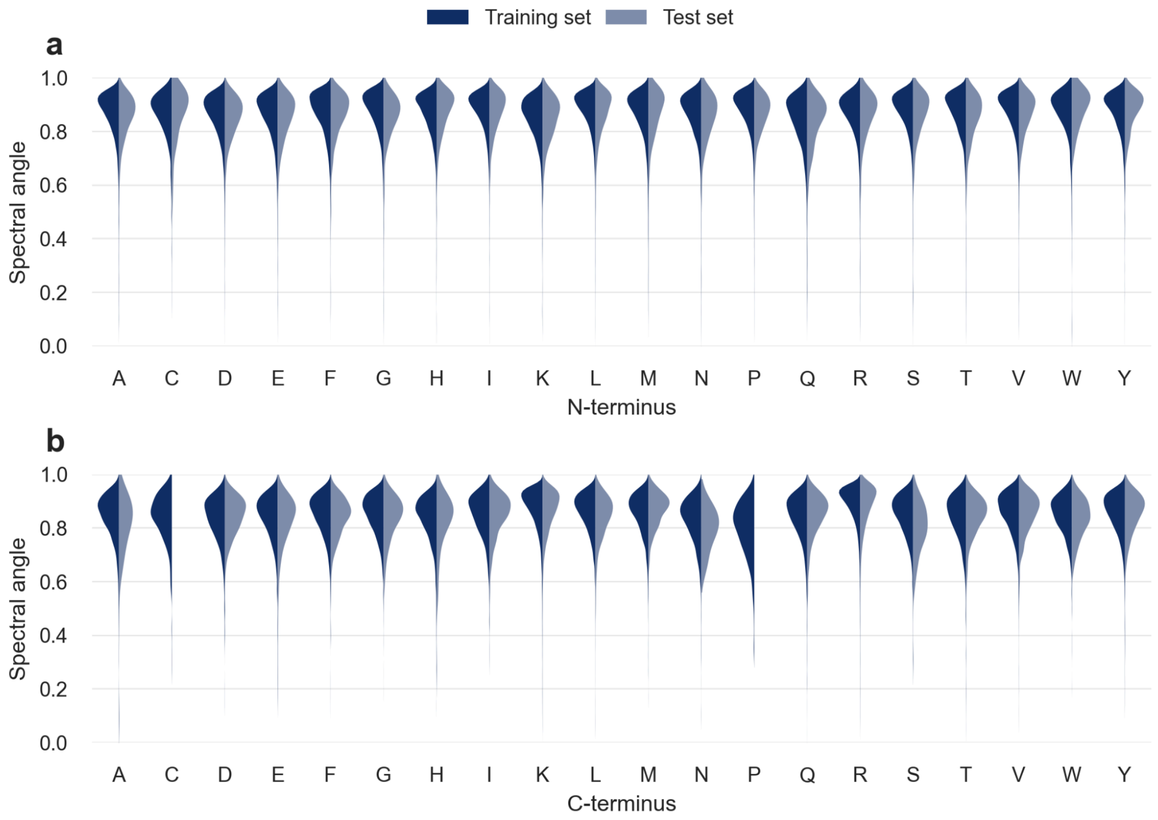

Supplementary Figure 4. The timsTOF Prosit 2023 model showed little bias for (**a**) N- and, (**b**) C-terminal amino acids. Source data are provided as a source data file.

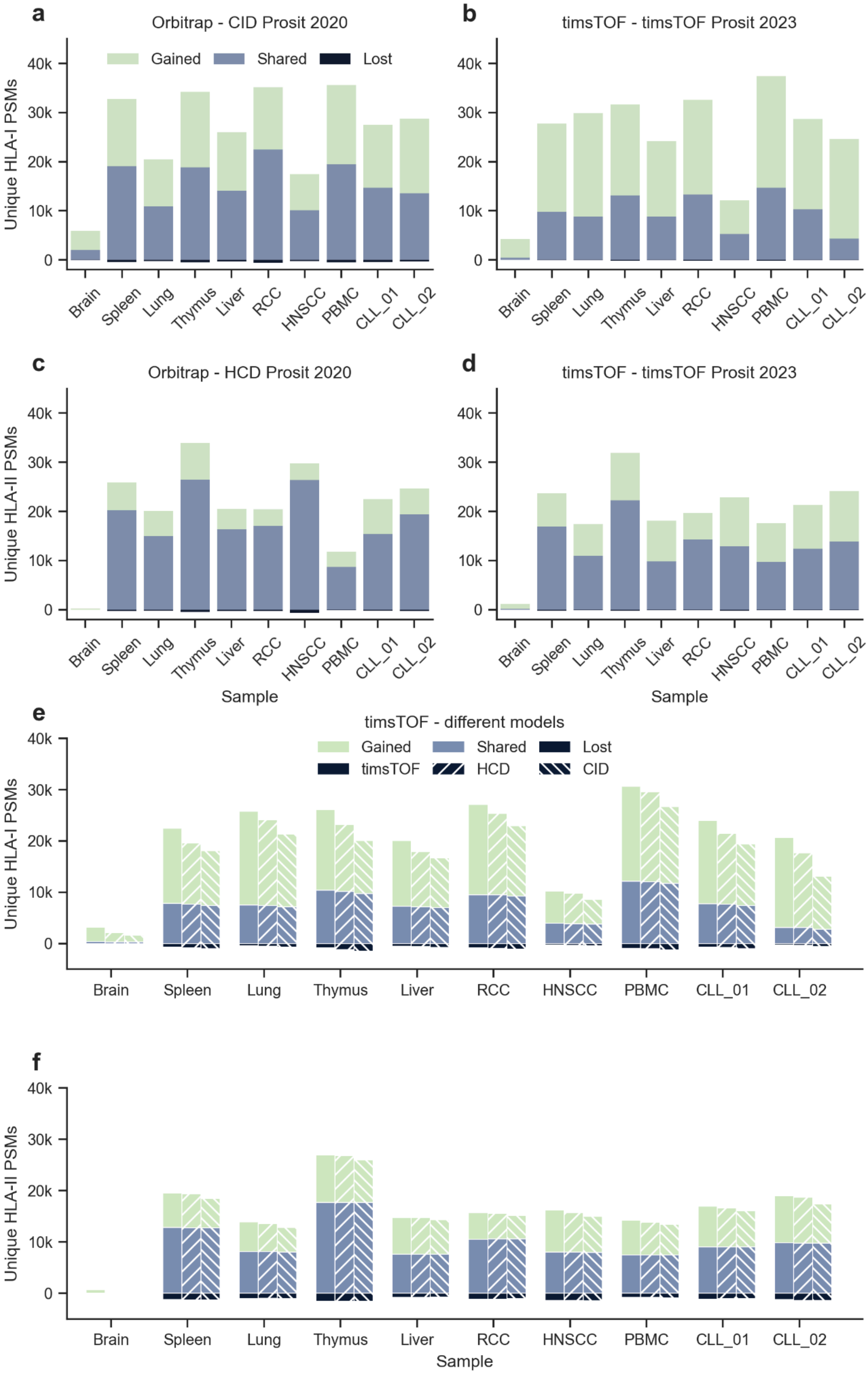

a
Orbitrap - CID Prosit 2020
Gained
Shared
Lost
b
timsTOF - timsTOF Prosit 2023
c
Orbitrap - HCD Prosit 2020
d
timsTOF - timsTOF Prosit 2023
e
timsTOF - different models
timsTOF
HCD
CID
f
Unique HLA-I PSMs
Unique HLA-II PSMs
Sample
Brain
Spleen
Lung
Thymus
Liver
RCC
HNSCC
PBMC
CLL_01
CLL_02
0
10k
20k
30k
40k

Supplementary Figure 5. Gained, shared, and lost identified PSMs for different sample types to compare PSM rescoring on Orbitrap data with PSM rescoring on timsTOF data. In general PSM rescoring was able to boost the number of PSMs, retaining true PSMs, gaining new PSMs, and losing only a small number of previously incorrect PSMs. **a** On average PSM rescoring of HLA-I Orbitrap data with the CID Prosit 2020 model resulted in a 1.9-fold increase. **b** On average PSM rescoring of HLA-I timsTOF data with the timsTOF Prosit 2023 model resulted in a 3.6-fold increase. **c** On average PSM rescoring of HLA-II Orbitrap data with the HCD Prosit 2020 model resulted in a 1.3-fold increase. **d** On average PSM rescoring of HLA-II timsTOF data with the timsTOF Prosit 2023 model resulted in a 2.3-fold increase. **e** To evaluate the effect of the fragment ion intensity prediction model on PSM rescoring, the RT prediction-based features were excluded. On average PSM rescoring of HLA-I timsTOF data with the timsTOF Prosit 2023 model resulted in a 3.4-fold increase. On average PSM rescoring of HLA-I timsTOF data with the HCD Prosit 2020 model resulted in a 2.9-fold increase. On average PSM rescoring of HLA-I timsTOF data with the CID Prosit 2020 model resulted in a 2.5-fold increase. **f** To evaluate the effect of the fragment ion intensity prediction model on PSM rescoring, the RT prediction-based features were excluded. On average PSM rescoring of HLA-II timsTOF data with the timsTOF Prosit 2023 model resulted in a 1.6-fold increase. On average PSM rescoring of HLA-II timsTOF data with the HCD Prosit 2020 model resulted in a 1.6-fold increase. On average PSM rescoring of HLA-II timsTOF data with the CID Prosit 2020 model resulted in a 1.5-fold increase. RCC = renal cell carcinoma; HNSCC = head and neck squamous-cell carcinoma; PBMC = peripheral blood mononuclear cell; CLL = chronic lymphocytic leukemia. Source data are provided as a source data file.

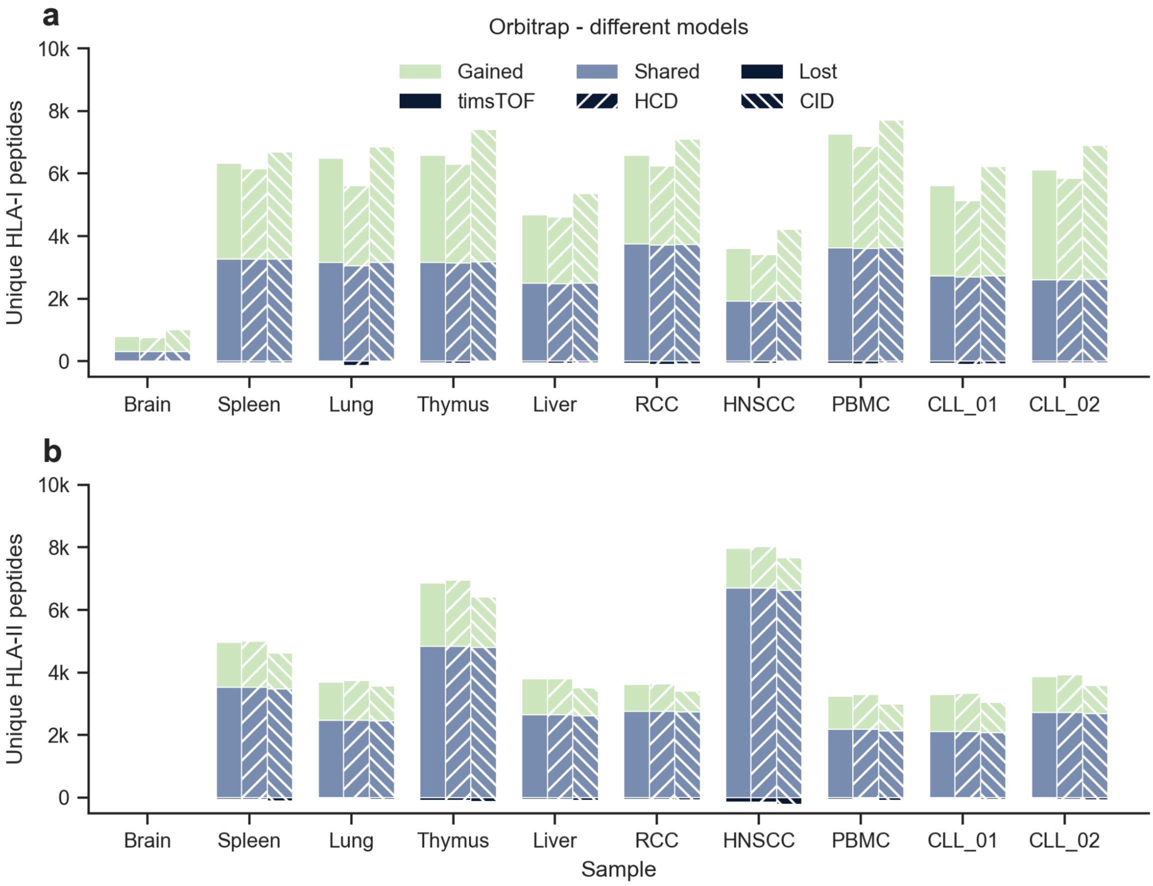

Supplementary Figure 6. Gained, shared, and lost peptide identifications for PSM rescoring with different fragment ion intensity prediction models. In general PSM rescoring was able to boost the number of PSMs, retaining true PSMs, gaining new PSMs, and losing only a small number of previously incorrect PSMs. **a** To evaluate the effect of the fragment ion intensity prediction model on PSM rescoring, the RT prediction-based features were excluded. On average PSM rescoring of HLA-I Orbitrap CID data with the timsTOF Prosit 2023 model resulted in a 2.0-fold increase. On average PSM rescoring of HLA-I timsTOF data with the HCD Prosit 2020 model resulted in a 1.9-fold increase. On average PSM rescoring of HLA-I timsTOF data with the CID Prosit 2020 model resulted in a 2.3-fold increase. **b** To evaluate the effect of the fragment ion intensity prediction model on PSM rescoring, the RT prediction-based features were excluded. On average PSM rescoring of HLA-II Orbitrap HCD data with the timsTOF Prosit 2023 model resulted in a 1.4-fold increase. On average PSM rescoring of HLA-II Orbitrap HCD data with the HCD Prosit 2020 model resulted in a 1.4-fold increase. On average PSM rescoring of HLA-II Orbitrap HCD data with the CID Prosit 2020 model resulted in a 1.3-fold increase. RCC = renal cell carcinoma; HNSCC = head and neck squamous-cell carcinoma; PBMC = peripheral blood mononuclear cell; CLL = chronic lymphocytic leukemia. Source data are provided as a source data file.

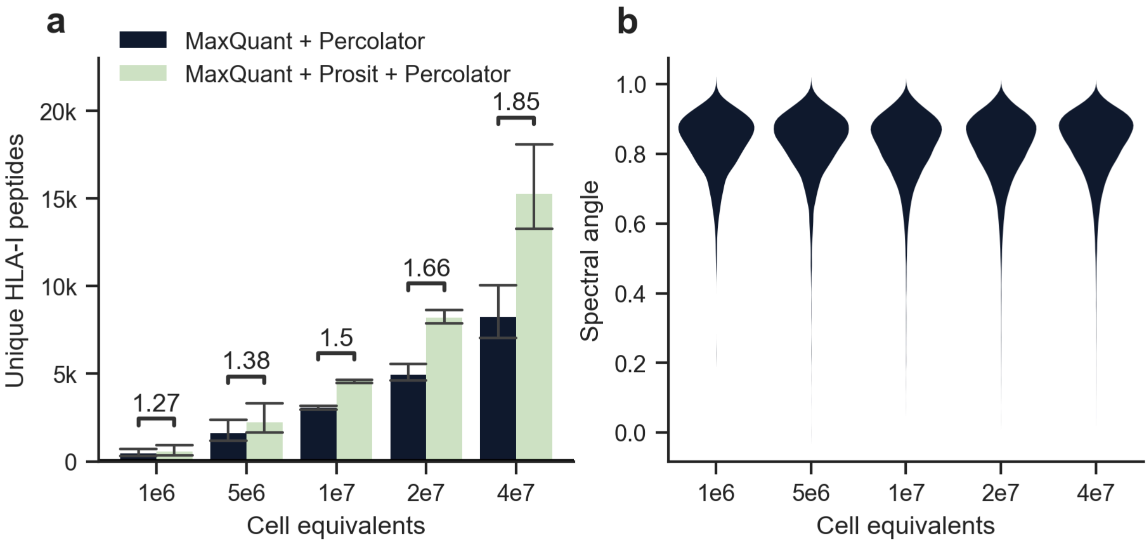

Supplementary Figure 7. a Bar chart illustrating the mean number of unique HLA-I peptides identified from 1 million to 40 million A-375 cell equivalents. The whiskers indicate the standard deviation and above each bar the fold change is shown between MaxQuant + Percolator and MaxQuant + Prosit + Percolator. **b** Violin plots depicting the normalized spectral contrast angle between predicted and observed spectra. The median spectral angle observed for cell equivalents from 1 million to 40 million, respectively, were 0.85, 0.85, 0.84, 0.84, and 0.85. Source data are provided as a source data file.

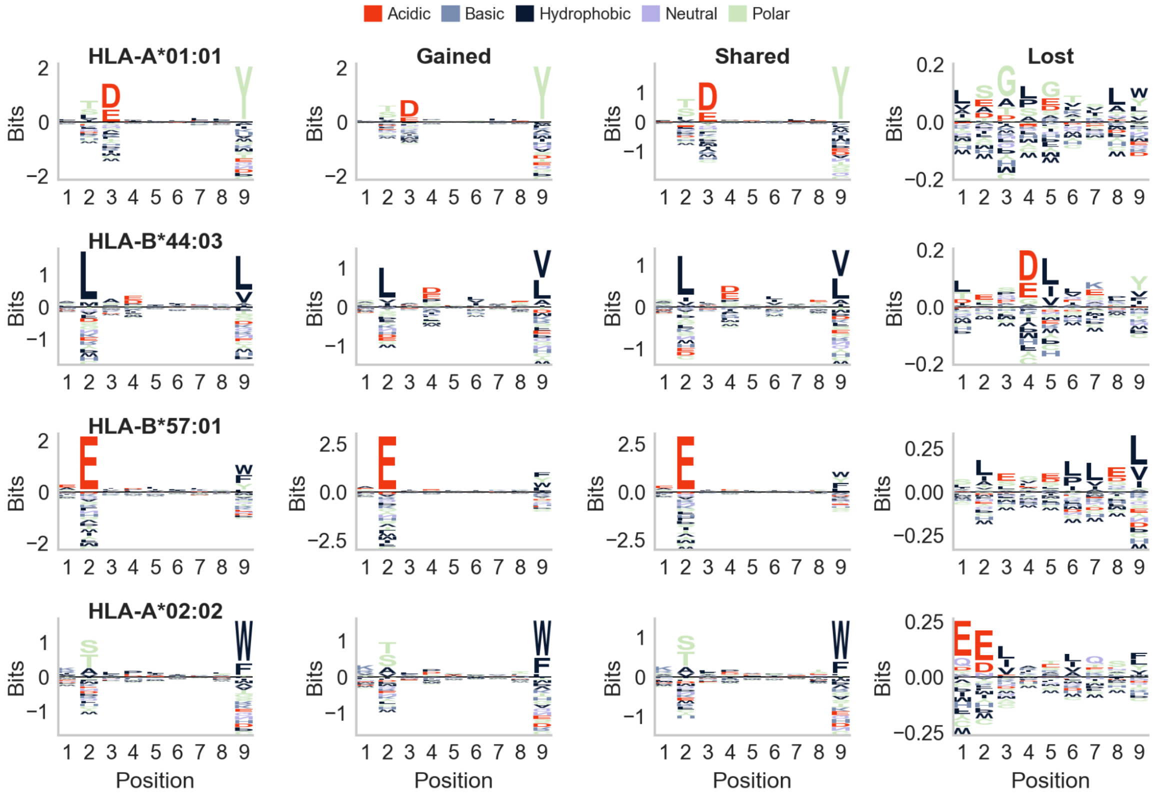

Supplementary Figure 8. HLA motif plots were generated for four monoallelic cell lines expressing specific HLA alleles: A*01:01 (1,406 unique peptides), A*02:02 (3,483 unique peptides), B*44:03 (989 unique peptides), and B*57:01 (1,510 unique peptides) [2]. From the PSM rescoring results of the 1 million to 40 million cell equivalents, a total of 16,641 unique gained peptides, 13,208 unique shared peptides, and 447 unique lost peptides were clustered, resulting in four peptide motifs for each peptide set. Next to each HLA motif, the peptide motif is plotted with the smallest Kullback-Leibler distance compared to the HLA motif. Amino acids are colored according to their physico-chemical properties (red acidic, blue basic, black hydrophobic, purple neutral, and green polar amino acids).

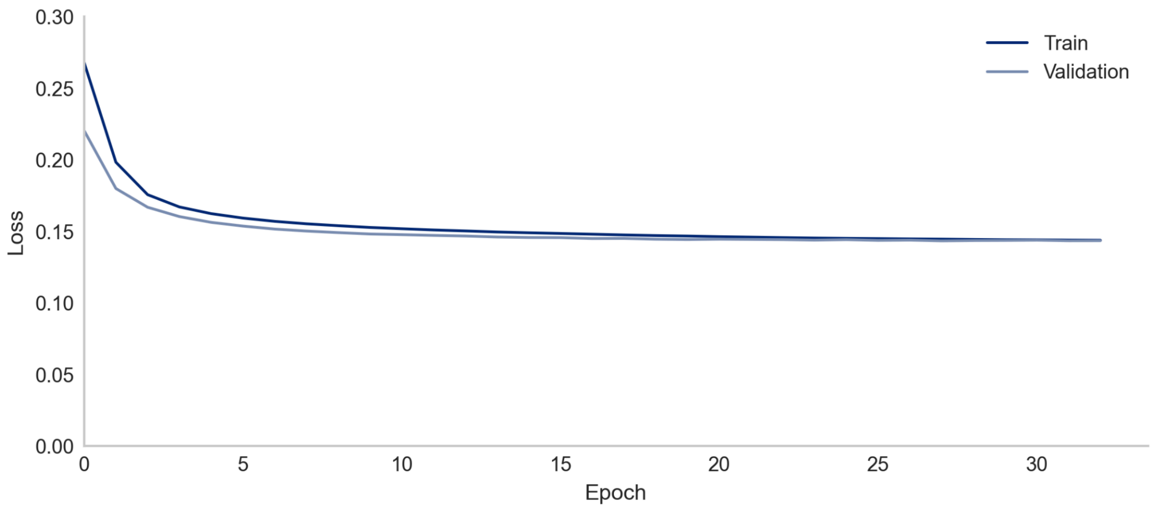

Supplementary Figure 9. Loss plot illustrating the model training progress. The loss function was 1 minus the normalized spectral contrast angle. The plot displays the train and validation loss values across the epochs, offering insights into the convergence and performance of model training over time.

Feature	Description
SpecId	Internal spectrum ID
Label	1 indicates target, -1 decoy
ScanNr	Scan number
filename	Raw file name
ExpMass	Fixed constant to allow PSMs with different theoretical mass to compete for the same PSM in target-decoy competition
CID	1 indicates CID fragmentation
Charge1	Boolean for charge state 1
Charge2	Boolean for charge state 2
Charge3	Boolean for charge state 3
Charge4	Boolean for charge state 4
Charge5	Boolean for charge state 5
Charge6	Boolean for charge state 6
HCD	1 indicates HCD fragmentation
KR	Number of K or R amino acids in sequence
Mass	Experimental mass
RT	Experimental retention time
UnknownFragmentationMethod	1 indicates unknown fragmentation method
abs_diff_Q1	Quantile 1 absolute differences between the predicted and theoretical ions
abs_diff_Q2	Quantile 2 absolute differences between the predicted and theoretical ions
abs_diff_Q3	Quantile 3 of the absolute differences between the predicted and theoretical ions
collision_energy_aligned	Selected aligned collision energy
cos	Cosine similarity on all potential y- and b-ions

count_not_observed_and_not_predicted	Number of theoretical ions not observed in the experimental spectrum nor in the predicted spectrum
count_not_observed_and_not_predicted_b	Number of theoretical b-ions not observed in the experimental spectrum nor found in the predicted spectrum
count_not_observed_and_not_predicted_y	Number of theoretical y-ions not observed in the experimental spectrum nor found in the predicted spectrum
count_not_observed_but_predicted	Number of theoretical ions not observed in the experimental spectrum but found in the predicted spectrum
count_not_observed_but_predicted_b	Number of theoretical b-ions not observed in the experimental spectrum but found in the predicted spectrum
count_not_observed_but_predicted_y	Number of theoretical y-ions not observed in the experimental spectrum but found in the predicted spectrum
count_observed	Number observed annotated ions in the experimental spectrum
count_observed_and_predicted	Number observed annotated ions in the experimental spectrum and found in the predicted spectrum
count_observed_and_predicted_b	Number observed annotated b-ions in the experimental spectrum and found in the predicted spectrum
count_observed_and_predicted_y	Number observed annotated y-ions in the experimental spectrum and found in the predicted spectrum
count_observed_b	Number observed annotated b-ions in the experimental spectrum
count_observed_but_not_predicted	Number observed annotated ions in the experimental spectrum and not found in the predicted spectrum
count_observed_but_not_predicted_b	Number observed annotated b-ions in the experimental spectrum and not found in the predicted spectrum
count_observed_but_not_predicted_y	Number observed annotated y-ions in the experimental spectrum and not found in the predicted spectrum

count_observed_y	Number observed annotated y-ions in the experimental spectrum
count_predicted	Number of the predicted ions
count_predicted_b	Number of the predicted b-ions
count_predicted_y	Number of the predicted y-ions
fraction_not_observed_and_not_predicted	Number of theoretical ions not observed in the experimental spectrum nor found in the predicted spectrum divided by the number of theoretical ions
fraction_not_observed_and_not_predicted_b	Number of theoretical b-ions not observed in the experimental spectrum nor found in the predicted spectrum divided by the number of theoretical b-ions
fraction_not_observed_and_not_predicted_b_vs_predicted_b	Number of theoretical b-ions not observed in the experimental spectrum nor found in the predicted spectrum divided by the number of b-ions found in the predicted spectrum
fraction_not_observed_and_not_predicted_vs_predicted	Number of theoretical ions not observed in the experimental spectrum nor found in the predicted spectrum divided by the number of ions found in the predicted spectrum
fraction_not_observed_and_not_predicted_y	Number of theoretical y-ions not observed in the experimental spectrum nor found in the predicted spectrum divided by the number of theoretical y-ions
fraction_not_observed_and_not_predicted_y_vs_predicted_y	Number of theoretical y-ions not observed in the experimental spectrum nor found in the predicted spectrum divided by the number of y-ions found in the predicted spectrum
fraction_not_observed_but_predicted	Number of theoretical ions not observed in the experimental spectrum but found in the predicted spectrum divided by the number of theoretical ions
fraction_not_observed_but_predicted_b	Number of theoretical b-ions not observed in the experimental spectrum but found in the predicted spectrum divided by the number of theoretical b-ions
fraction_not_observed_but_predicted_b_vs_predicted	Number of theoretical b-ions not observed in the experimental spectrum but found in the predicted spectrum divided by the number of b-ions found in the predicted spectrum

fraction_not_observed_but_predicted_vs_predicted	Number of theoretical ions not observed in the experimental spectrum but found in the predicted spectrum divided by the number of ions found in the predicted spectrum
fraction_not_observed_but_predicted_y	Number of theoretical y-ions not observed in the experimental spectrum but found in the predicted spectrum divided by the number of theoretical y-ions
fraction_not_observed_but_predicted_y_vs_predicted	Number of theoretical y-ions not observed in the experimental spectrum but found in the predicted spectrum divided by the number of y-ions found in the predicted spectrum
fraction_observed	Number of theoretical ions observed in the experimental spectrum divided by the number of theoretical ions
fraction_observed_and_predicted	Number of theoretical ions observed in the experimental spectrum and found in the predicted spectrum divided by the number of theoretical ions
fraction_observed_and_predicted_b	Number of theoretical b-ions observed in the experimental spectrum and found in the predicted spectrum divided by the number of theoretical b-ions
fraction_observed_and_predicted_b_vs_predicted_b	Number of theoretical b-ions observed in the experimental spectrum and found in the predicted spectrum divided by the number of predicted b-ions
fraction_observed_and_predicted_vs_predicted	Number of theoretical ions observed in the experimental spectrum and found in the predicted spectrum divided by the number of predicted ions
fraction_observed_and_predicted_y	Number of theoretical y-ions observed in the experimental spectrum and found in the predicted spectrum divided by the number of theoretical y-ions
fraction_observed_and_predicted_y_vs_predicted_y	Number of theoretical y-ions observed in the experimental spectrum and found in the predicted spectrum divided by the number of predicted y-ions
fraction_observed_b	Number of theoretical b-ions observed in the experimental spectrum divided by the number of theoretical b-ions

fraction_observed_but_not_predicted	Number of theoretical ions observed in the experimental spectrum but not found in the predicted spectrum divided by the number of theoretical ions
fraction_observed_but_not_predicted_b	Number of theoretical b-ions observed in the experimental spectrum but not found in the predicted spectrum divided by the number of theoretical b-ions
fraction_observed_but_not_predicted_b_vs_predicted_b	Number of theoretical b-ions observed in the experimental spectrum but not found in the predicted spectrum divided by the number of predicted b-ions
fraction_observed_but_not_predicted_vs_predicted	Number of theoretical ions observed in the experimental spectrum but not found in the predicted spectrum divided by the number of predicted ions
fraction_observed_but_not_predicted_y	Number of theoretical y-ions observed in the experimental spectrum but not found in the predicted spectrum divided by the number of theoretical y-ions
fraction_observed_but_not_predicted_y_vs_predicted_y	Number of theoretical y-ions observed in the experimental spectrum but not found in the predicted spectrum divided by the number of predicted y-ions
fraction_observed_y	Number of theoretical y-ions observed in the experimental spectrum divided by the number of theoretical y-ions
fraction_predicted	Number of theoretical ions found in the predicted spectrum divided by the number of theoretical ions
fraction_predicted_b	Number of theoretical b-ions found in the predicted spectrum divided by the number of theoretical b-ions
fraction_predicted_y	Number of theoretical y-ions found in the predicted spectrum divided by the number of theoretical y-ions
max_abs_diff	Max absolute difference between the predicted and theoretical ions
mean_abs_diff	Mean absolute difference between the predicted and theoretical ions

min_abs_diff	Minimum absolute difference between the predicted and theoretical ions
missedCleavages	Number of missed cleavages
modified_cosine	Modified cosine similarity [3] on all potential y- and b-ions
mse	Mean square error
pearson_corr	Pearson correlation on all potential y- and b-ions
pearson_corr_b_ions	Pearson correlation on b-ions
pearson_corr_double_charge	Pearson correlation on doubly charged ions
pearson_corr_single_charge	Pearson correlation on singly charged ions
pearson_corr_triple_charge	Pearson correlation on triply charged ions
pearson_corr_y_ions	Pearson correlation on y-ions
sequence_length	Peptide sequence length
spearman_corr	Spearman correlation on all potential y- and b-ions
spearman_corr_b_ions	Spearman correlation on b-ions
spearman_corr_double_charge	Spearman correlation on doubly charged ions
spearman_corr_single_charge	Spearman correlation on singly charged ions
spearman_corr_triple_charge	Spearman correlation on triply charged ions
spearman_corr_y_ions	Spearman correlation on y-ions
spectral_angle	Normalized spectral contrast angle (SA) on all potential y- and b-ions
spectral_angle_b_ions	Normalized spectral contrast angle (SA) on b-ions
spectral_angle_double_charge	Normalized spectral contrast angle (SA) on doubly charged ions
spectral_angle_single_charge	Normalized spectral contrast angle (SA) on singly charged ions
spectral_angle_triple_charge	Normalized spectral contrast angle (SA) on triply charged ions
spectral_angle_y_ions	Normalized spectral contrast angle (SA) on y-ions
spectral_entropy_similarity	Spectral entropy similarity [4] on all potential y- and b-ions

std_abs_diff	Standard deviation of the absolute differences between retention time and aligned predicted retention time
abs_rt_diff	Absolute difference between retention time and aligned predicted retention time
lda_scores	Score returned by a linear discriminant analysis on the spectral angle to estimate false discovery rates
pred_RT	Predicted aligned retention time
iRT	Predicted indexed retention time
Peptide	Peptide sequence
Protein	Protein description

Supplementary Table 1. List of features used for PSM rescoring with Prosit predictions. Both the features and the descriptions are reported. When fragment ion intensity prediction models were compared, the following features were removed: abs_rt_diff, lda_scores, pred_RT, and iRT.

Feature	Description
SpecId	Internal spectrum ID
Label	1 indicates target, -1 decoy
ScanNr	Scan number hash
filename	Raw file name
ExpMass	Fixed constant to allow PSMs with different theoretical mass to compete for the same PSM in target-decoy competition
CID	1 indicates CID fragmentation
Charge1	Boolean for charge state 1
Charge2	Boolean for charge state 2
Charge3	Boolean for charge state 3
Charge4	Boolean for charge state 4
Charge5	Boolean for charge state 5
Charge6	Boolean for charge state 6
HCD	1 indicates HCD fragmentation
KR	Number of K or R amino acids in sequence
Mass	Experimental mass
UnknownFragmentationMethod	1 indicates unknown fragmentation method
andromeda	Score returned by the search engine
andromeda_delta_score	Delta score from the best next peptide identification returned by the search engine
missedCleavages	Number of missed cleavages
sequence_length	Peptide sequence length
Peptide	Peptide sequence
Protein	Protein description

Supplementary Table 2. List of features used for PSM rescoring without Prosit predictions. Both the Features and the descriptions are reported. These features were used to postprocess the MaxQuant results to have a fair comparison with the

References

1. Wilhelm, M. *et al.* Deep learning boosts sensitivity of mass spectrometry-based immunopeptidomics. *Nat. Commun.* 12, 3346 (2021).
2. Sarkizova, S. *et al.* A large peptidome dataset improves HLA class I epitope prediction across most of the human population. *Nat. Biotechnol.* 38, 199–209 (2020).
3. McGann, C. D. *et al.* Real-Time Spectral Library Matching for Sample Multiplexed Quantitative Proteomics. *J. Proteome Res.* 22, 2836–2846 (2023).
4. Li, Y. *et al.* Spectral entropy outperforms MS/MS dot product similarity for small-molecule compound identification. *Nat. Methods* 18, 1524–1531 (2021).
